# Emergence and dissemination of equine-like G3P[8] rotavirus A in Brazil between 2015 and 2021

**DOI:** 10.1128/spectrum.03709-23

**Published:** 2024-03-07

**Authors:** Meylin Bautista Gutierrez, Ighor Arantes, Gonzalo Bello, Lúcia Helena Berto, Leonardo Hermes Dutra, Rodrigo Bentes Kato, Tulio Machado Fumian

**Affiliations:** 1 Laboratório de Virologia Comparada e Ambiental, Instituto Oswaldo Cruz, Rio de Janeiro, Brazil; 2 Laboratório de Arbovírus e Vírus Hemorrágicos, Instituto Oswaldo Cruz, Fiocruz, Rio de Janeiro, Brazil; 3 Coordenação Geral de Laboratórios de Saúde Pública, Ministério da Saúde, Brasília, Brazil; Changchun Veterinary Research Institute, Chinese Academy of Agricultural Sciences, Changchun, China

**Keywords:** rotavirus, phylogeography, Brazil, Bayesian analysis

## Abstract

Rotavirus A (RVA) is a major cause of acute gastroenteritis globally that is classically genotyped by its two immunodominant outer capsid proteins, VP7 (G-) and VP4 (P-). Recent evidence suggests that the reassortant equine-like G3P[8] strain played a substantial role in RVA transmission in Brazil since 2015. To understand its global emergence and dissemination in Brazilian territory, stool samples collected from 11 Brazilian states (*n* = 919) were genotyped by RT-qPCR and proceeded to sequence the VP7 gene (*n* = 102, 79 being newly generated) of the G3P[8] samples with pronounced viral loads. Our phylogenetic genotyping showed that G3P[8] became the dominant strain in Brazil between 2017 and 2020, with equine-like variants representing 75%–100% of VP7 samples in this period. A Bayesian discrete phylogeographic analysis strongly suggests that the equine-like G3P[8] strain originated in Asia during the early 2010s and subsequently spread to Europe, the Caribbean, and South America. Multiple introductions were detected in Brazil between 2014 and 2017, resulting in five national clusters. The reconstruction of the effective population size of the largest Brazilian cluster showed an expansion until 2017, followed by a plateau phase until 2019 and subsequent contraction. Our study also supports that most mutations fixed during equine-like G3P[8] evolution were synonymous, suggesting that adaptive evolution was not an important driving force during viral dissemination in humans, potentially increasing its susceptibility to acquired immunity. This research emphasizes the need for comprehensive rotavirus genomic surveillance that allows close monitoring of its ever-shifting composition and informs more effective public health policies.

**IMPORTANCE:**

Our original article demonstrated the origin and spread in a short time of equine-like G3P[8] in Brazil and the world. Due to its segmented genome, it allows numerous mechanisms including genetic drift and reassortment contribute substantially to the genetic diversity of rotavirus. Although the effectiveness and increasing implementation of vaccination have not been questioned, a matter of concern is its impact on the emergence of escape mutants or even the spread of unusual strains of zoonotic transmission that could drive epidemic patterns worldwide. This research emphasizes the need for comprehensive rotavirus genomic surveillance, which could facilitate the formulation of public policies aimed at preventing and mitigating its transmission.

## INTRODUCTION

**Fig 1:**
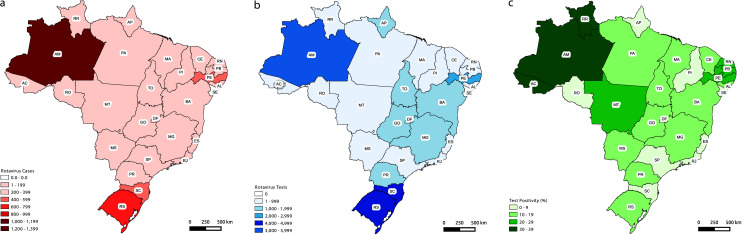
Rotavirus dissemination in Brazil. (a) Total cases, (b) test positivity, and (c) rotavirus tests performed in Brazil according to data obtained by the Laboratory Environment Manager (Gerenciador de Ambiente Laboratorial, GAL)/Brazilian Ministry of Health.

Rotavirus, a genus in the *Sedoreoviridae* family (1), consists of non-enveloped viruses with a double-stranded RNA genome (2). Among these, species A rotavirus (RVA) is a major culprit responsible for acute gastroenteritis (AGE) in humans, posing a significant global health threat, especially among children under 5 years old in resource-limited countries (3). In Brazil, between 2013 and 2023, more than 5,000 severe RVA infection cases have been officially documented, with the highest concentration observed in states of the northern region (41%), followed by the southern (26%), northeastern (17%), southeastern (9%), and central-Western (5%) regions (Fig. 1a), although with severe heterogeneity in positivity and number of tests performed (Fig. 1b and c). Globally, in 2016 alone, RVA was associated with an estimated ~130,000 deaths (4). To combat this public health challenge, live-attenuated oral rotavirus vaccines such as Rotarix and RotaTeq have been integrated into national immunization programs for over 15 years now (5). As of January 2023, 123 countries have successfully incorporated RVA vaccines into their national immunization programs (6), leading to a substantial reduction in RVA-related hospitalizations and fatalities. This remarkable effort averted approximately 80,000 deaths worldwide in 2016 (4, 7), underscoring the vital role these vaccines play in safeguarding public health.

The infectious RVA virion exhibits a non-enveloped icosahedral structure with a 100 nm of diameter (8). This species is characterized by a triple-layered capsid and harbors a genome comprising 11 double-stranded RNA (dsRNA) segments, which encode 6 viral structural proteins (VP1–VP4, VP6, and VP7) and 6 non-structural ones (NSP1–NSP6) (2). RVA can be classified into 42G and 58P genotypes, based on the genotyping of the two immunodominant outer capsid proteins: VP7 (G‐type) and VP4 (P‐type) (9). Nevertheless, despite the plethora of potential permutations between these genotypes, a restricted repertoire of configurations is responsible for the majority of human infections, namely, G1P[8], G2P[4], G3P[8], G4P[8], G9P[8], and G12P[8] (Dóró et al., 2014). Furthermore, an assessment of all 11 genomic segments reveals that the majority of human RVA detections can be allocated to three specific genomic constellations. These constellations include (i) the Wa-like/genogroup 1 (G1/3/4/9/12P[8]-I1-R1-C1-M1-A1-N1-T1-E1-H1), (ii) DS-1-like/genogroup 2 (G2-P[4]-I2-R2-C2-M2- A2-N2-T2-E2-H2), and (iii) the less prevalent AU-1-like/genogroup 3 (G3-P[9]-I3- R3-C3-M3-A3-N3-T3-E3-H3). Each of these constellations is believed to have originated from a distinct animal species (10, 11). As a segmented virus, genome reassortment represents a pivotal mechanism for generating diversity, which can effectively evade the immune responses of both partially and fully susceptible individuals. This phenomenon, arising from co-infected hosts, therefore, plays a critical role in counteracting immune recognition (12).

Over the past decade, a variant of rotavirus (G3) with a specific reassortment event has gained significant attention in the ongoing global circulation of RVA. This event involved the emergence of the equine-like G3P[8] DS-1-like RVA strain, which was initially identified in Australia and Thailand in 2013 (13, 14). Subsequently, it has been reported in various regions, including Asia, Australia, Europe, and the Americas (15–28). In Brazil, the equine-like G3P[8] DS-1-like strain was first detected in 2015 when it was isolated from a 10-year-old unvaccinated female displaying AGE symptoms in the southern state of Paraná (18). In the following year, an additional instance of its detection occurred in the northern state of Amazonas (17). Subsequently, a comprehensive study conducted by our group revealed its dominance and widespread presence across the country a few years after the first detection events (24). Indeed, in 2018 and 2019, it accounted for prevalence rates of 84% and 65%, respectively, among all genotyped samples (24). The most likely scenario for the emergence of this G3P[8] DS-1-like genotype involves an intergenogroup reassortment mechanism. This mechanism occurred between co-circulating DS-1-like G1P[8] strains, initially identified in children with severe AGE in Japan in 2012 (29–31), and equine-like G3P[4] strains, which were also isolated in children with acute diarrhea in Sendai, Japan, in 2013 and are considered prototypes (23, 32). Notably, these equine-like strains share a similar viral structure with equine strains, suggesting an early reassortment event involving RVA/Horse-wt/IND/Erv105/2003–2005/G3P[X]/DQ981479.1. This strain was initially isolated from fecal samples of an Indian foal (23, 32, 33) and circulating human G2P[4] strains (13). More recently, an intergenogroup reassortment event birthed the equine-like G3P[8] Wa-like (34) and circulation of G3P[8] Wa-Like was reported (28, 35, 36).

A growing body of evidence suggests that the equine-like G3P[8] strain played a substantial role in the dissemination of RVA in Brazil from 2015 to 2019 (24, 37). The worldwide circulation of variant equine G3 was driven by multiple strains possessing either Wa-like or DS-1-like constellations (28, 36, 38) although there is evidence that the majority of human infections caused by equine-like G3P[8] are associated with a genogroup DS-1-like backbone genes (13, 15–18, 20–22, 25, 39–41). However, the precise structure and spatiotemporal dynamics of equine-like G3P[8] spread remain unclear. This study aims to elucidate the emergence and dissemination patterns of the equine-like G3P[8] strain, on a national and global scale.

## METHODOLOGY

### Samples and ethics aspects

This study included 919 samples, collected between 2014 and 2021, from stool specimens of children and adults with symptoms of AGE. The samples entered the AGE surveillance routine performed by the Laboratory of Comparative and Environmental Virology (LVCA) at Fiocruz, Rio de Janeiro (an integral part of the structure of the Ministry of Health) by medical requests. All samples were accompanied by their clinical-epidemiological records. Procedures were approved by the Ethics Committee of the Oswaldo Cruz Foundation (FIOCRUZ), Brazil (Approval number: CAAE 94144918.3.0000.5248), and conducted according to the guidelines of the Declaration of Helsinki. Stool samples were manipulated anonymously and patient data were maintained securely in compliance with the Ethical Protocol Statement ISO 15189. Patient-informed consent was waived by the Fiocruz Ethical Committee, and patients’ data were maintained anonymously and securely.

### RNA extraction, RT-PCR amplification, and genotyping

Viral RNA was extracted from 140 µL of supernatant using QIAamp Viral RNA Mini kit (QIAGEN, Valencia, CA, USA). A TaqMan-based quantitative one-step RT-PCR was used for viral detection and quantification, using primers and probes targeting the conserved NSP3 gene segment were used as previously described (42). The RVA-positive samples obtained by RT-qPCR were G- and P-genotyped using a one-step multiplex RT-PCR. The reactions were performed using the Qiagen One Step RT-PCR kit (Qiagen), using forward conserved primers VP7uF or VP4uF. In this study, primers for the G3 type of G and P[8] type of P (43–45) were both used. The G- and P- genotypes were assigned based on different amplicon sizes base pairs (bp) using agarose gel analysis.

### G3P[8] VP7 sequencing

Samples (i) genotyped as G3P[8] and (ii) exhibiting cycle threshold (Ct) values indicatives of pronounced viral load (<30) were selected for posterior Sanger sequencing of their VP7 gene, using consensus primers of Beng9/End9 (43, 46). The generated amplicon fragments (1,062 bp) were purified using the ExoSAP clean-up kit (ThermoFisher Scientific, Waltham, Ma, USA) or the QIAquick Gel Extraction Kit (Qiagen, Foster City, CA, USA) and sent to the FIOCRUZ Institutional Platform for DNA sequencing (PDTIS). Chromatogram analysis and RVA consensus sequences were obtained using BioEdit 7.7.1 Sequence Alignment Editor and Geneious Prime 2021.1.1 (Biomatters Ltd, Auckland, New Zealand).

### RVA G3P[8] phylogenetic classification

To identify RVA equine-like G3P[8] VP7 sequences, a data set composed of sequences (i) generated in this study and (ii) publicly available at GenBank (47) up to 31 December 2022 (*n* = 578) was genotyped in a maximum likelihood (ML) statistical framework alongside reference sequences of VP7 lineages I-IX (*n* = 49) (10, 11, 14, 33, 48–51). The data set was aligned using MAFFT v7.467 (52), manually curated when necessary, and subjected to an ML phylogenetic analysis using IQ-TREE v2.1.2 (53), under the nucleotide substitution model found best fitted to our data as selected by jModelTest2 v.2.1.10 (54) software. The approximate likelihood-ratio test assessed the branch support based on the Shimodaira–Hasegawa-like procedure (SH-aLRT) (55) with 1,000 replicates. In the reconstructed topology, sequences were genotyped by their clustering with reference sequences with significant statistical support (aLRT > 90%).

### Temporal signal

Sequences of the VP7 gene assigned to the RVA equine-like G3P[8] strain were investigated regarding the existence of a temporal structure, a prerequisite for the subsequent time-scaled phylogeographic analysis. These samples were selected and subjected to an ML analysis with previously described parameters. The temporal structure of this phylogenetic tree was assessed by a regression analysis of the root-to-tip divergence against sampling time using TempEst v1.5.36 (56). Sequences that diverged more than 1.5 interquartile ranges from the root-to-tip regression were considered outliers and removed from the analysis. The significance of the association between the two variables was assessed with a Spearman correlation test implemented in the R programming language v.4.1.2 (R Core Team, 2022).

### Bayesian time-scaled phylogeographic analysis

The time of the most recent common ancestor (*T*_MRCA_), the evolutionary rate, and the phylogeographic dispersion pattern of the RVA equine-like G3P[8] cluster were jointly reconstructed in a Bayesian framework using BEAST v1.10 (57, 58) software package, with BEAGLE (59) to improve run-time. A time-scaled phylogeny was inferred using (i) the coalescent Bayesian skyline (BSKL) coalescent (60) tree prior, (ii) the nucleotide substitution model found best fitted to our data as selected by jModelTest2 v.2.1.10 (54) software, and (iii) the molecular clock found best fitted to our data by the log marginal likelihood estimation based on the stepping-stone (61) and path-sampling (62) methods. Migration events were modeled using a reversible discrete phylogeographic model (63) with a CTMC rate reference prior (64). The number of location transitions (viral migrations between locations) throughout evolutionary history was estimated using Markov jump counts (65). Geographic locations were assigned to the sequences, based on their accompanying metadata. To obtain a more detailed reconstruction of RVA equine-like G3 dissemination in Brazilian territory, the geographic coding of the country’s sequences was based on their state of origin, while foreign ones were grouped by continental regions. The Markov Chain Monte Carlo (MCMC) chains were run for 200 × 106 generations and convergence and uncertainty of parameter estimates were assessed by calculating the Effective Sample Size (ESS) and 95% Highest Probability Density (HPD) values, respectively, after excluding the initial 10% of each run with Tracer v1.7.1 (66). The convergence of parameters was considered when ESS ≥ 200. The maximum clade credibility tree was summarized with TreeAnnotator v1.10.4 (58) and visualized using treeio v3.1.7 (67) and ggtree v3.2.1 (68).

### Reconstruction of ancestral sequences

To elucidate the amino acid changes that became fixed during the evolutionary process of equine-like G3P[8] dissemination in Brazil, the reconstruction of both the ancestral sequences for the five Brazilian clusters (BR-I-V) and the complete cluster was performed. This reconstruction was accomplished using the Bayesian MCMC approach, implemented in BEAST v1.10 (57, 58). Following the exclusion of sequences associated with the burn-in phase, the remaining sequences were employed to generate a consensus sequence for each Most Recent Common Ancestor (MRCA) through the utilization of Seaview v.4 (69).

### Bayesian demographic dynamics of equine-like G3P[8] in Brazil

The evolution across time of the effective sample size (*N*_e_) of RVA equine-like G3 Brazilian clusters with dimension (*n* ≥ 30) that allowed for more accurate modeling was done with the coalescent-based Bayesian Skygrid model (BSKG) (70) implemented in BEAST v1.10 (57, 66). The posterior distribution of values for the root height of the clusters inferred previously during the phylogeographic analysis was used as an informative prior. The Bayesian analysis was conducted with the same parameters previously described.

## RESULTS

### Rotavirus A G3P[8] detection in Brazil between 2014 and 2021

**Fig 2:**
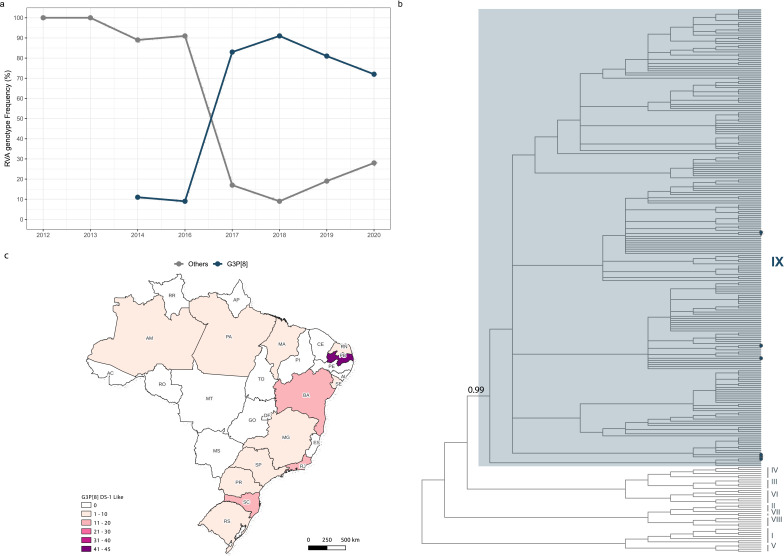
Rotavirus Equine-like G3P[8]dissemination in Brazil. (a) Genotyping by RT-qPCR of stool samples (*n* = 919) submitted from 11 Brazilian states to the LVCA at Fiocruz, Rio de Janeiro. (b) Genotyping of publicly available (*n* = 578) and newly generated (*n* = 102) VP7 sequences by a ML tree inferred with reference sequences of G3P[8] lineages I-IX (*n* = 49). The lineage IX (equine-like) cluster is highlighted in light gray, and its reference sequences are indicated by large blue tips. The statistical support (aLRT) of the lineage IX cluster is indicated in the tree. For improved clarity, sequences classified outside the lineage IX cluster were removed. (c) Distribution of G3P[8] sequences classified as equine-like G3P[8] across all Brazilian states. Maps were generated with QGIS v.3.10.2 software (http://qgis.org) using public access data downloaded from the GADM v.3.6 database (https://gadm.org) and shapefiles obtained from the Brazilian Institute of Geography and Statistics (https://portaldemapas.ibge.gov.br/portal.php#homepage).

Between 2014 and 2021, this study collected 919 RVA-positive stool specimens from 11 Brazilian states (Table S1). Utilizing RT-qPCR for G- and P- genotyping, the analysis assigned approximately 40% of these samples to the G3P[8] genotype (Fig. 2a). During the three initial years (2014–2016), G3P[8] was a minority genotype, comprising only 10% of the samples (*n*_TOTAL_ = 53). However, from 2017 to 2020, it became the predominant one, constituting around 80% of the samples (*n*_TOTAL_ = 317). Despite keeping this majority position until 2020, its prevalence fell to approximately 70% by that year, after reaching a peak in 2018 (~90%). From this point onward, the limited number of samples received (*n*_TOTAL_ = 5) precluded additional inferences.

**TABLE 1:** RVA equine-like G3P[8] VP7 data set^*a*^

Location		Complete data set (*n* = 190)	Newly generated (*n* = 79)	Previously published (*n* = 111)	Sampling range
Asia		51	–	51	2013–2020
Brazil (N)	Amazonas	9	–	9	2016
Brazil (NE)	Bahia	11	11	–	2017–2020
	Maranhão	3	3	–	2016–2019
	Paraíba	2	2	–	2018
	Pernambuco	40	40	–	2017–2021
	Rio Grande do Norte	1	1	–	2019
	Sergipe	1	1	–	2019
Brazil (SE)	Rio de Janeiro	11	6	5	2016–2020
	Minas Gerais	1	1	–	2017
	São Paulo	2	2	–	2018
Brazil (S)	Paraná	7	1	6	2015–2016
	Rio Grande do Sul	5	5	–	2016–2019
	Santa Catarina	6	6	–	2018–2020
Caribbean		15	–	15	2014–2016
Europe		23	–	23	2015–2019
North America		1	–	1	2015
South America		1	–	1	2015

^*a*^ The table details the VP7 sequences assigned to the equine-like G3P[8]. Sequences were either generated by this study or previously published. All entries in the table are stratified by location of sampling and are accompanied by their sampling time range. Brazilian sequences are additionally classified according to their state of origin. N, north; NE, northeast; SE, southeast; S, south.

### G3P[8] VP7 sequence generation and genotyping

To understand equine-like G3P[8] contribution to Brazil’s G3P[8] dissemination, samples that fitted the established criteria (G3P[8] genotype/Ct < 30) were selected for Sanger sequencing of their VP7 gene. Subsequently, VP7 sequences (i) previously published (*n* = 578) until 31 March 2023, (ii) newly produced (*n* = 102), and (iii) genotypic reference sequences of lineages I-IX (*n* = 49) were combined and proceeded to genotype the query sequences in this data set (765 nts, from position 79 to 844 of reference sequence YP_002302222.1) based on their clustering with the reference ones in a maximum likelihood topology (Fig. 2b and c). In accordance with the samples assigned by this analysis to the equine-like strain (lineage IX, *n* = 209, 36%), its earliest detection harkens back to 2013 and, in the 2013–2020 period, represents a significant fraction of all available G3P[8] VP7 sequences (~40%), having become the majority in 2017 (>50%). Asia was the site of the first detections, followed by the Caribbean in 2014, and by Europe, North America, and South America, in 2015. Since 2013, the regions with the highest overall relative equine-like G3P[8] prevalence were the Caribbean (94%), followed by Europe (61%) and Asia (24%). Little to no samples (~0%) were retrieved from Africa, the Middle east, North America, Oceania, or South America. In Brazil, the oldest sequence in our data set came from the southern state of Paraná, collected in 2015 (18), followed by a spatial spread sampling in the following year. In fact, in 2015–2020, the majority (75%–100%) of G3P[8] detections in the country belonged to the equine-like strain.

### Temporal structure of RVA equine-like G3P[8] VP7 data set

**Fig 3:**
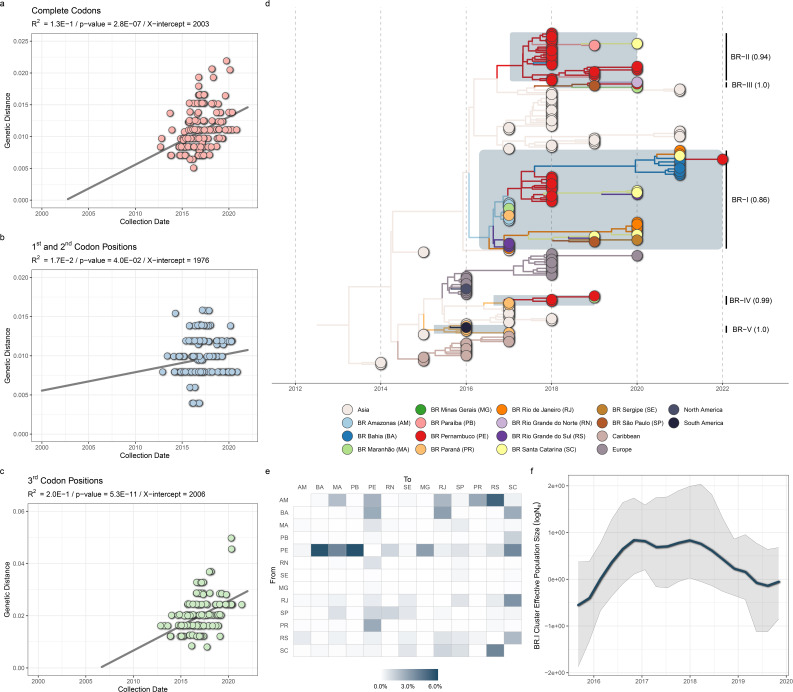
Equine-like G3P[8] evolution. (a–c) Time-divergence regression of equine-like G3P[8] sequences by means of their complete (**a**), first and second (**b**), and third codon positions (**c**). (d) Time-scaled phylogeographic reconstruction of equine-like G3P[8] sequences. The tree is colored according to the inferred location of its internal nodes, utilizing the color code on the bottom of the panel. Posterior probability support of Brazilian clusters (BR-I-V) is indicated alongside the cluster name. (e) Viral migrations between Brazilian states, measured by a Markov Jumps count. (f) Temporal evolution of the effective population size (*N*_e_) of the BR-I cluster inferred by the coalescent-base BSKG. The graph was plotted until the time of the last coalescent event detected in the tree the analysis is based.

After the remotion of some outlier sequences (*n* = 19), the maximum likelihood topology inferred with the equine-like G3P[8] data set (*n* = 190) (Table 1) showed a significant (*P* = 2.8E−07, X-intercept date = 2006) association between collection dates and root-to-tip divergence (Fig. 3a), prompting us to utilize the observed temporal structure to infer divergence times across the evolution of the lineage. To gain deeper insights into the prevailing class of mutations contributing to the observed temporal structure of equine-like G3P[8], the same analysis was performed using only (i) the first and second (non-synonymous substitutions) and (ii) the third (synonymous substitutions) codon positions. The temporal structure was statistically kept in both scenarios (*P*-value < 0.05), indicating the accumulation of both synonymous and non-synonymous mutations within the equine-like G3P[8] cluster over time. Nevertheless, there is evidence to suggest that synonymous mutations constitute the predominant class of mutations that have become fixed within the cluster, as both (i) the recovered *T*_MRCA_ in the regression performed with third codon position mutations showed a significantly smaller gap (3 years) to the same parameter recovered in the analysis of complete codons, than the gap observed in the regression performed with first and second codon positions mutations (27 years) and (ii) the *P*-value observed in the analysis with only the third position (5.3E−11) was pronouncedly smaller than the same parameter observed in the analysis of the first two positions (4.0E−02) (Fig. 3b and c).

### RVA equine-like G3P[8] global emergence and early dissemination

The evolution rate, the time to the most recent common ancestor (*T*_MRCA_), and the spatial dynamics of RVA equine-like G3P[8] were jointly estimated with the BSKL model (60). Between a strict and an uncorrelated relaxed molecular clock, the former was considered best fitted to our data (Table S2) and used in subsequent analysis. The posterior distribution of the evolution rate estimated (median value = 3.8 × 10^−3^ substitutions/site/year, 95% HPD: 2.9 × 10^−3^–4.9 × 10^−3^) fell within boundaries found by similar analysis using the same subgenomic region of RVA (71–74). The MMC Tree (Fig. 3d) obtained with these parameters showed a clear source-sink structure, with its root in Asia (posterior state probability, PSP = 100%) in the early 2010 s (median value = 2012, 95% HPD: 2011–2012), and posterior seeding of clusters in Europe, Caribbean, and Brazil. Unlike the Brazilian clusters, the ones established in Europe and the Caribbean had low statistical support (posterior probability < 25%) and should be considered with caution in the current data set.

**TABLE 2:** Spatio-temporal dynamics of RVA equine-like G3P[8] emergence in Brazil^*a*^

Cluster	*n*	Posterior probability	*T*_MRCA_ (95% HPD)	Sampling range	Location	Posterior state probability
BR-I	58	0.86	2015 (2015–2016)	2016–2021	Amazonas	0.70
BR-II	28	0.94	2016 (2015–2016)	2017–2019	Pernambuco	0.99
BR-III	6	1.0	2016 (2015–2016)	2016–2018	Paraná	0.86
BR-IV	5	0.99	2014 (2015–2016)	2015–2016	Paraná	0.96
BR-V	4	1.0	2017 (2015–2016)	2018–2019	São Paulo	0.33

^*a*^ The table details the Brazilian RVA equine-like G3P[8] clusters identified by a Bayesian discrete phylogeographic analysis. HPD, highest probability density.

### RVA equine-like G3P[8] emergence in Brazil

The available data set of RVA equine-like G3P[8] supports the existence of multiple introductions from Asia (*n* = 5) into the Brazilian territory in a short span of around 5 years, between 2014 (95% HPD: 2014 to 2015) and 2017 (95% HPD: 2017 to 2018) (Table 2). All clusters generated by these migrations had significant posterior values (>85%), and aggregated distinct fractions of the Brazilian sequences (*n* = 99), from BR-I (58%) to BR-V (4%). From 2020, all sequences were assigned to the BR-I cluster, while the other four clusters had their most recent samples detected from 2016 (BR-IV) to 2019 (BR-II and BR-V). The phylogeographic analysis supports the most probable location of these clusters MRCAs (most recent common ancestors) as located in the country’s northern (BR-I, Amazonas), northeastern (BR-II, Pernambuco), southern (BR-III and BR-IV, Paraná), and southeastern (BR-V, São Paulo) regions. These locations were mostly supported by significant posterior state probability values (PSP > 85%), except for BR-I (70%) and BR-V (33%). To understand the molecular composition of these clusters, the ancestral sequences of each were reconstructed. Overall, in relation to the equine-like G3P[8] VP7 MRCA sequence, seven positions with synonymous mutations were found, most of them in the ancestor of BR-III (~60%), and only two non-synonymous ones, all of them also in the ancestor of BR-III.

### RVA equine-like G3P[8] dissemination in Brazil

The BR-I cluster stood out as the main route of RVA equine-like G3P8 dissemination in the country, as it was the only one that aggregates sequences of all four Brazilian regions analyzed, with major contributions from the states of Bahia (northeastern, 18%); Amazonas (north, 16%); Pernambuco (northeastern, 12%); and Rio de Janeiro (southeastern, 11%) (Table 3). BR-II, BR-III, and BR-V, conversely, were mainly detected in the country’s northeastern region (97%, 67%, and 75%, respectively), with contributions from multiple of its states. BR-IV (the cluster with the oldest MRCA in the country) had a more restricted area of dissemination and was composed only of previously published (18) sequences from the southern state of Paraná, with an additional detection in South America (34). Across all four Brazilian clusters, this was the only viral migration to a foreign territory detected. The northern state of Amazonas and the northeastern Pernambuco, appointed as the root of the two main clusters of equine-like G3P[8] dissemination in the country, stood out as the main sites of outward virus migrations inside the country, as measured by a Markov Jumps count (Fig. 3e). While the main destinies of migrations from Amazonas were states in different regions of the country (Rio Grande do Sul, 32% Pernambuco, 17%, Rio de Janeiro, 15%), most of Pernambuco’s emitted migrations were to other states in the same northeastern region (Paraíba, 22%; Bahia, 21%; Maranhão, 15%).

**TABLE 3:** Spatial composition of RVA equine-like G3P[8] Brazilian clusters^*a*^

Location		BR-I (*n* = 58)	BR-II (*n* = 28)	BR-III (*n* = 6)	BR-IV (*n* = 5)	BR-V (*n* = 4)
Brazil (N)	Amazonas	9	–	–	–	–
Brazil (NE)	Bahia	10	1	–	–	–
	Maranhão	1	–	1	–	1
	Paraíba	–	2	–	–	–
	Pernambuco	12	24	3	–	1
	Rio Grande do Norte	–	–	–	–	1
	Sergipe	1	–	–	–	–
Brazil (SE)	Rio de Janeiro	11	–	–	–	–
	Minas Gerais	1	–	–	–	–
	São Paulo	1	–	–	–	1
Brazil (S)	Paraná	1	–	2	4	–
	Rio Grande do Sul	5	–	–	–	–
	Santa Catarina	5	1	–	–	–
South America		–	–	–	1	–

^*a*^ The table details the spatial composition of each of the five equine-like G3P[8] Brazilian clusters identified. N, north; NE, northeast; SE, southeast; S, south.

### Equine-like G3P[8] demographic dynamics in Brazil

To reconstruct the temporal evolution of the effective population size (*N*_e_) of equine-like G3P[8] as it spread in Brazil, the coalescent-based Bayesian Skygrid model (70) implemented in Beast 1.10 (57, 58) was utilized. The reconstruction was restricted to the BR-I cluster (*n* = 58) (Fig. 3f), given the very limited dimension of clusters BR-II-V (*n* < 25). In BR-I history, the analysis revealed at least three distinct stages in the *N*_e_ dimension: (i) an increase from its emergence in 2015 until late 2016, (ii) stabilization from 2016 until 2018, and (iii) a subsequent decrease from 2018 until late 2019.

## DISCUSSION

In this study, the evolution of RVA equine-like G3P[8] across multiple continents as told by its VP7 gene was reconstructed. In Brazil, it was possible to investigate these dynamics in particular detail, given the new sequences produced here. Currently, the most probable hypothesis gives an account of equine-like G3P[8] emerging in the early 2010s in Asia and, in the span of around five years, being introduced into Europe, North America, South America, and the Caribbean. In Brazil, the initial detection of equine-like G3P[8] dates back to 2015, followed by the establishment of multiple clusters in the following 2 years, becoming the majority genotype in the country in the 2017/2019 period, with a reduction in prevalence registered afterward.

Our data support the occurrence of multiple introductions of equine-like G3P[8] RVA strains from Asia into South America, Europe, and the Caribbean. All five introductions into South America were strongly supported and occurred in Brazil, leading to the formation of the five national clusters (BR-I-V). It is important to acknowledge that the limited global sampling of RVA sequences may have obscured intermediate steps in the dissemination process. This limitation could potentially result in the amalgamation of all introductions to a common root node located in Asia. Notably, some continental and subcontinental regions, including North America, South America, Africa, and the Middle East, were significantly underrepresented or entirely absent from our data set despite documented instances of equine-like G3P[8] strain detections (35, 75). Expanding the scope of sampling efforts could prove immensely beneficial, particularly for countries and regions grappling with a higher burden of RVA dissemination. Enhanced sampling has the potential to yield more robust phylogeographic reconstructions, enabling the testing of hypotheses about the principal drivers behind the strain’s establishment.

Equine-like G3P[8] introductions into Brazil (*n* = 5) were widespread in the territory and birthed local clusters in states located in the country’s northern (BR-I), northeastern (BR-II), southeastern (BR-V), and southern regions (BR-III/IV). After its first introduction in 2015 (BR-III), the equine-like G3P[8] displaced the G2P[4] genotype that previously dominated the Brazilian RVA dissemination in the space of three years. In our phylogeographic analysis, three of the local clusters (BR-II/III/IV) had their state of origin highly supported (posterior state probability, PSP > 85%), while the remaining two presented low PSP values in their root nodes, BR-I hypothetically originated in Amazonas (70%), and BR-V, in São Paulo (34%). Despite this lack of more robust support for Amazonas to be located at the root of BR-I, the largest cluster in the country, socio-epidemiological evidence seems to support a centrality for the state in the country’s RVA endemic dissemination. Amazonas houses (i) the majority of RVA-positive cases in the country (*n* = 1,284 cases between 2013 and 2023, ~25% of the cases in the country) (Fig. 1a); (ii) the second highest Rotavirus test positivity rate (33%) (Fig. 1b), behind only the also northern state of Roraima (36%); and (iii) one of the smallest fraction of its citizens with access to wastewater treatment in the country (13.8%) (IBGE, 2022).

Once established, these five clusters (BR-I/V) gave rise to transmission networks with distinct dimensions, from BR-I/II (*n* = 58 to 28) to BR-III/IV/V (*n* = 6 to 4). Noticeably, by 2020, all clusters except BR-I were probably on the verge of potential extinction, given their non-existent sampling. Considering the hypothesis that a differential accumulation of mutations in an immunodominant protein such as VP7 could influence viral fitness for its impact on the efficacy of pre-existing immunity, the MRCA sequences of the complete equine-like G3P[8] genotype and the BR-I/IV clusters were reconstructing. Notably, apart from two non-synonymous mutations identified in the BR-III cluster, the ancestors of the other four Brazilian clusters exhibited complete amino acid identity. Consequently, the observed disparities in their dissemination across the country could not be ascribed to virological factors within their VP7 protein, but to either proteins encoded by their other 10 remaining genomic segments, epidemiological characteristics of the populations in which their dissemination occurred, or even to stochastic factors. For instance, it is noteworthy the fact that the two largest clusters in the country, BR-I and BR-II, evolved from introductions into two states with some of the highest numbers of Rotavirus cases detected in the country (Amazon and Pernambuco, respectively), therefore potentially contributing with a substantial number of new infections to both transmission clusters, granting them an advantage in their spread within the country. As a reflection of Amazonas and Pernambuco’s location at the root of both clusters, half (~50%) of equine-like G3P[8] viral migrations in the country originated in one of the two states.

The *N*_e_ reconstruction of the BR-I cluster revealed a dynamic pattern characterized by rapid expansion between 2015 and 2017, followed by a plateau phase lasting approximately 1 year and, subsequently, a sustained contraction up to the present. Interestingly, our analyses suggest that synonymous mutations constitute the predominant class of mutations that have become fixed within the equine-like G3P[8] genotype. When the temporal fluctuation of the *N*_e_ of BR-I cluster is considered in the context of mostly synonymous mutations being accumulated during the genotype evolution, it brings the hypothesis that the absence of a more robust antigenic evolution, as seen in other RNA viruses (76), may render the equine-like G3P[8] group more vulnerable to population-level immunity. This vulnerability can eventually bring the displacement of the strain in the next few years, a possibility already hinted at by our prevalence analysis. The limited extent of non-synonymous evolution observed here within the equine-like G3P[8] genotype remains a phenomenon not yet fully understood. However, investigating Rotavirus mutational tolerance, a feature more comprehensively studied in other models (77), may offer valuable insights into the evolutionary mechanisms of Rotavirus, as exemplified in this study by the equine-like G3P[8] strain.

A significant constraint in this study undoubtedly stems from the limited data availability, encompassing both insufficient new data points and incomplete genomes of the samples under examination. The varying positivity rates observed across Brazilian states, along with the absence of samples from countries known to exhibit equine-like G3P[8] cases, strongly underscore the issue of undersampling that has affected our analysis. In typical phylogeographic analyses, undersampling specific regions can serve as a strategy to construct a data set that accurately reflects the epidemiological landscape of the regions in question, potentially leading to more plausible results. However, in our case, practical constraints already hindered the full implementation of this strategy. For instance, Brazilian states with a high number of cases detected, such as Amazonas (AM, north) and Rio Grande do Sul (RS, south), are underrepresented in our data set due to the limited number of available sequences. Furthermore, the absence of complete genomes imposes substantial limitations on the scope of our analysis, hindering our ability to gain a deeper understanding of genotype dissemination within the country and rendering our study blind to potential new recombination events.

In conclusion, this study represents the first phylogeographical study of Rotavirus in Brazil, a crucial undertaking given the substantial burden this pathogen places on the nation’s population. Despite the inherent limitations in sequence availability, our research has revealed the establishment of equine-like G3P[8] strains in the country, rooted in multiple introductions spanning Brazilian territory, resulting in clusters characterized by heterogeneous spatial distribution patterns. Moreover, our findings suggest that an increase in population immunity may be limiting the cluster dissemination, potentially leading to its displacement. These insights underscore the urgent need for comprehensive Rotavirus genomic surveillance in Brazil. Such surveillance can serve as an indispensable tool for closely monitoring the dynamic nature of its dissemination and facilitating the development of more efficacious public health policies.

## Data Availability

Publicly available data sets were analyzed in this study. This data can be found here: GenBank accession numbers: OR675060 to OR675138. This study is registered in the Brazilian National System for Genetic Heritage and Associated Traditional Knowledge Management (SisGen, No. A837EB6).

## SUPPLEMENTAL MATERIAL

Tables S1 and S2 spectrum.03709-23-s0001.docx Table S1 describes the number of genotyped samples over the years. Table S2 describes the models used to analyze the data set.

ASM does not own the copyrights to Supplemental Material that may be linked to, or accessed through, an article. The authors have granted ASM a non-exclusive, world-wide license to publish the Supplemental Material files. Please contact the corresponding author directly for reuse.
