# Supplementary material for: Emergence and dissemination of equine-like G3P[8] rotavirus A in Brazil between 2015 and 2021
Source: Microbiol Spectr. 2024 Mar 7;12(4):e03709-23. doi: 10.1128/spectrum.03709-23 (PMC10986506; doi:10.1128/spectrum.03709-23)
Supplement: Tables S1 and S2 — Table S1 describes the number of genotyped samples over the years. Table S2 describes the models used to analyze the data set. [file spectrum.03709-23-s0001.docx]

**SUPPLEMENTARY TABLES**

**Supplementary Table 1:** G- and P- Genotyping of RVA Positive Stool Samples

| **Location** | | **2014**  **(n = 323)** | | **2016**  **(n = 209)** | | **2017**  **(n = 165)** | | **2018**  **(n = 86)** | | **2019**  **(n = 84)** | | **2020**  **(n = 47)** | | **2021**  **(n = 5)** | |
| --- | --- | --- | --- | --- | --- | --- | --- | --- | --- | --- | --- | --- | --- | --- | --- |
|  |  | **G3P[8]** | **Others** | **G3P[8]** | **Others** | **G3P[8]** | **Others** | **G3P[8]** | **Others** | **G3P[8]** | **Others** | **G3P[8]** | **Others** | **G3P[8]** | **Others** |
| Brazil (N) | Amazonas | - | - | - | - | - | - | - | - | 1 | - | - | - | - | - |
| Brazil (NE) | Bahia | - | - | - | - | - | - | - | - | - | - | 20 | 4 | 1 | 1 |
|  | Maranhão | 13 | 2 | - | 15 | 2 | 8 | 8 | 1 | 1 | - | - | - | - | - |
|  | Paraíba | - | - | - | - | - | - | 10 | - | - | - | - | - | - | - |
|  | Pernambuco | 5 | 73 | 1 | 52 | 108 | 9 | 19 | 6 | 24 | 5 | 2 | - | 1 | 1 |
|  | Sergipe | 1 | 13 | 2 | 13 | 6 | 6 | 4 | - | 3 | - | - | - | - | - |
| Brazil (SE) | Espírito Santo | - | 8 | 1 | - | - | - | 1 | - | 1 | - | - | - | - | - |
|  | Rio de Janeiro | 2 | 9 | 1 | 1 | - | - | - | - | 9 | - | 7 | 3 | - | - |
|  | Minas Gerais | 5 | 51 | 1 | 9 | 8 | - | 2 | - | - | - | - | - | - | 1 |
| Brazil (S) | Rio Grande do Sul | 2 | 62 | 10 | 35 | 7 | 1 | 13 | 1 | 16 | 11 | 1 | 3 | - | - |
|  | Santa Catarina | 6 | 71 | 3 | 65 | 6 | 4 | 21 | - | 13 | - | 4 | 3 | - | - |

*The table details the G- and P- genotyping of 919 RVA positive stool samples (an integral part of the structure of the Ministry of Health) by medical requests collected between 2014 and 2021. The samples took part in the AGE surveillance routine performed by the Laboratory of Comparative and Environmental Virology (LVCA) at Fiocruz, Rio de Janeiro.*

**Supplementary Table 2:** Best Fit Molecular Clock Model for the RVA equine-like G3P[8] VP7 dataset

|  | **Strict** | **UCLD** | **Models**  **Compared** | **Log BF** |
| --- | --- | --- | --- | --- |
| **Stepping Stone Sampling log ml** | -4798 | -4793 | UCLD/Strict | 5 |
| **Path Sampling**  **log ml** | -4795 | -4790 | UCLD/Strict | 5 |

*Log marginal likelihood (ml) estimates for the strict and uncorrelated relaxed lognormal distributed molecular clock models obtained using the generalized stepping-stone sampling and path sampling methods. The Log Bayes factor (BF) is the difference of the log ml between alternative (H1) and null (H0) models (H1/H0). Log BFs > 3 indicates that model H1 is more strongly supported by the data than model H0.*
